# Psychosocial Correlates of Suicidal Behavior among Adolescents under Confinement Due to the COVID-19 Pandemic in Aguascalientes, Mexico: A Cross-Sectional Population Survey

**DOI:** 10.3390/ijerph18094977

**Published:** 2021-05-07

**Authors:** Alicia Edith Hermosillo-de-la-Torre, Stephania Montserrat Arteaga-de-Luna, Denise Liliana Acevedo-Rojas, Angélica Juárez-Loya, José Alberto Jiménez-Tapia, Francisco Javier Pedroza-Cabrera, Catalina González-Forteza, Manuel Cano, Fernando A. Wagner

**Affiliations:** 1 Psychology Department, Universidad Autónoma de Aguascalientes [Autonomous University of Aguascalientes], Aguascalientes 20131, Mexico; alicia.hermosillo@edu.uaa.mx (A.E.H.-d.-l.-T.); stephania.arteaga@edu.uaa (S.M.A.-d.-L.); denise.acevedo@edu.uaa.mx (D.L.A.-R.); francisco_pedroza@hotmail.com (F.J.P.-C.); 2 Clinical and Health Psychology Department, Psychology Faculty, Universidad Nacional Autónoma de México (UNAM), Mexico City 04510, Mexico; ajuarezloya@comunidad.unam.mx; 3 Instituto Nacional de Psiquiatría [National Institute of Psychiatry], Tlalpan 14370, Mexico; alberj@imp.edu.mx (J.A.J.-T.); catiartes@gmail.com (C.G.-F.); 4 Department of Social Work, University of Texas at San Antonio, San Antonio, TX 78207, USA; manuel.cano@utsa.edu; 5 School of Social Work, University of Maryland Baltimore, Baltimore, MD 21201, USA

**Keywords:** adolescents, suicidal behavior, psychosocial correlates, COVID-19

## Abstract

*Background*: Suicide and suicidal behaviors were already a global public health problem, producing preventable injuries and deaths. This issue may worsen due to the COVID-19 pandemic and may differentially affect vulnerable groups in the population, including children, adolescents, and young adults. The current study evaluated the association of affective variables (depression, hopelessness, and anxiety), drug use (alcohol, tobacco, and others), emotional intelligence, and attachment with suicidal behaviors. *Methods*: A state-wide survey included 8033 students (51% female, 49% male; mean age of 16 years) from science and technology high-schools using a standardized questionnaire that was distributed online. Multinomial logistic regression models tested associations between suicidal behaviors and several covariates. The analyses accommodated the complex structure of the sample. *Results*: Approximately 21% of all students reported a suicidal behavior (11% with a low-lethality suicide attempt, 6% with self-injuries, and 4% with a high-lethality suicide attempt). Variables associated with higher odds of suicidal behavior included: female sex, depression, hopelessness, anxiety, alcohol and tobacco use, childhood trauma, and having to self-rely as issues affecting attachment, and low self-esteem. Security of attachment was associated with lower odds of suicidal behavior. *Conclusions*: The complexity of suicidal behavior makes it clear that comprehensive programs need to be implemented.

## 1. Introduction

The COVID-19 pandemic is a worldwide public health challenge with a profound impact on the general population’s mental health, and particularly severe consequences for young people [1]. COVID-19′s negative effects may include a higher prevalence of suicidal behaviors (involving self-harm, low and high lethality attempts) [2], and secondary repercussions of social distancing and confinement. Some studies have reported that psychosocial and mental health contributory factors related to suicidal thoughts and behaviors may have increased during the outbreak [3].

Suicide and suicidal behaviors were already a global public health problem [4] as one of the most lethal and potentially preventable health issues [5], producing injuries and deaths that could be averted with appropriate information and strategies [6]. However, data suggest that this issue may worsen due to the pandemic and may differentially affect vulnerable groups in the population, including children, adolescents, and young adults [7]. Pre-COVID-19 data estimated that between 9 and 20% of adolescents and young adults have had a suicide attempt [8,9,10,11]. Suicide is the second leading cause of death in young people; it has been estimated that almost one million people die by suicide annually, that there are 20 suicide attempts for every death, and that it is a more frequent problem in low and middle-income countries [4,12,13].

Suicidal behavior includes suicidal ideation, as thoughts about ending one’s life [14]; suicide attempts refer to any self-injurious act with the definite intention to cause one’s own death, and death by suicide when a person ends his or her life. Suicidal ideation and suicide attempt have been associated with different psychosocial correlates. Negative affect, hopelessness, low self-esteem [15], drug use, and early life traumatic experiences have been found to increase the risk of suicidal behavior [16]. We also know that SARS-Cov2 interacts with biological processes implicated in suicidal behavior [17]. There is evidence that some adolescents who exhibit suicidal ideation proceed to attempt suicide within two years [18], and that they meet diagnostic criteria for a mental disorder [19]. It has also been reported that female adolescents are more likely to attempt, yet males are more likely to die by suicide, and that associated psychosocial correlates are present in both groups [20].

The COVID-19 crisis has increased concerns regarding suicide and suicidal behaviors, yet there is a lack of empirical evidence for low-income countries with poor health infrastructures [3]. Mexico is one such country, and data on the complex impact of COVID-19 on suicide and suicidal behavior is currently limited. Suicide and suicidal behaviors have continually increased in Mexico; the current suicide rate is 5.4 per 100,000 persons, with 34% of suicides corresponding to young people aged 18–29 years and 10% to adolescents and children aged 10–17 years [21,22]. Moreover, suicide rates more than doubled in the state of Aguascalientes over the past 10 years, reaching 10 cases per 100,000 persons in 2017, according to the latest available figures [23].

A mental health and suicidal behavior problem of alarming dimensions may be emerging [14,24]; therefore, it is necessary to investigate, which factors are related to the risk of suicide behaviors in Mexican adolescents, and even more so in the face of the health contingency. A recent study in Aguascalientes assessed several psychosocial constructs and highlighted that major depressive episode, low self-esteem, and the use of two or more drugs in the past month were associated with high-lethality cases of suicide attempt when controlling for several other covariates [25]. The present study aimed to expand that model for suicidal behaviors with three new design features: first, the current sample includes adolescents confined during COVID-19; second, the data are from a survey including all science and technology public high schools in Aguascalientes; and, third, several additional psychosocial constructs were added to the data collection instrument, as described in the following section. The current study further evaluated the association of emotional variables (depression, hopelessness, and anxiety), drug use (alcohol, tobacco, and others), emotional intelligence, and attachment with suicidal behaviors.

## 2. Materials and Methods

### 2.1. Study Design, Setting, and Population

The study “Variables Psicosociales Implicadas en el Desarrollo de Adolescentes y Jóvenes, VIDA-J [Psychosocial Variables Implicated in Adolescents’ and Youth’s Development]” is an observational, cross-sectional survey that was conducted from 9 November 2020, to 10 December 2020, with 8033 Mexican adolescents who were confined due to the COVID-19 pandemic and lived in the state of Aguascalientes, Mexico. All of them participated voluntarily after providing informed consent. Although this is a cross-sectional study that provides a current view of the situation, there are no available data that would allow us to establish whether the situation has changed due to the COVID-19 pandemic.

Approximately 51% of the participants self-identified as female and 49% as male; the age range was 14–21 with a mean of 16 and a standard deviation of 0.98. Data collection was done through an electronic questionnaire that was distributed using Google forms. Participants were at the end of their 1st, 3rd, and 5th high school grades through distance learning. In accordance with the authorities of the Instituto de Educación de Aguascalientes (Aguascalientes Institute of Education), the only criterion for inclusion was being an active student in any of the 32 schools of the Colegio de Estudios Científicos y Tecnológicos (High School for Scientific and Technological Studies), which belongs to the state’s public secondary education system. Based on the laws and regulations in force and the agreements of the Declaration of Helsinki [26], the informed consent of the school authorities served as a proxy for the adolescents’ parents’ consent.

### 2.2. Constructs and Measurements

#### 2.2.1. Suicidal Behaviors Schedule

The main outcomes of the present study were suicidal behaviors. Our conceptualization of suicidal behaviors as a categorical construct recognizes qualitative differences between self-injury, low-lethality suicidal behaviors, and high-lethality suicidal behaviors and is supported by prior research that identified four latent classes when analyzing suicidal behavior and associated psychosocial factors among adolescents in Campeche, Mexico [27,28,29]. A recent study also used a latent class approach and, although different populations and instruments were used, a four-class solution was identified as well [30]. Our research strategy was to compare youth with no suicidal behaviors to those with different levels of behaviors.

The suicidal behaviors schedule (Cédula de Conductas Suicidas (CCS)) was used as a screening instrument to ascertain self-inflicted injuries and suicidal attempts [27]. The initial questions inquired whether participants had ever hurt, cut, intoxicated, or caused harm to themselves to take their life away. It also included questions about the number of times; the age of the first and last time; the purpose, motivation, methods employed, and the use of mental health services (in open-ended questions); and the lethality indicator of these behaviors (i.e., the desire to die by suicide). The combination of data on suicidal behavior with the lethality indicator provides information to better understand and characterize the nature of the only or the latest suicidal behavior as either self-injurious behavior with the definite desire to continue living (hereafter referred to as self-injuries), an attempt with no definite intention to die (hereafter referred to as low-lethality suicide attempt), or an attempt with definite desire to die (hereafter referred to as high-lethality suicide attempt.) The schedule has demonstrated its concurrent validity in numerous studies with Mexican adolescent populations: concordant, with variables such as depression, drug use, and impulsivity; and, divergent, with family relationships, self-esteem, and internal locus of control, which are variables related to suicidal behavior [25,27,31].

#### 2.2.2. Questionnaire of Attachment Evaluation CAMIR-R

The questionnaire assesses attachment representations through past and present experiences and family functioning. There is a Spanish Short Version [32] and an adapted form [33] It examines secure and insecure attachment based on basic primary and secondary attachment strategies [34] using an interpretation guide [35]. We used the 32-item adolescent short version that uses a 5-point Likert scale response format (1 = strongly disagree to 5 = strongly agree). The questionnaire covers 7 factors: (1) Security: availability and support of attachment figures; this subscale measures the perception of feeling and being loved, trusting and knowing that when they need it, they can count on the attachment figures (items 3, 6, 7, 11, 13, 21, and 30). (2) Family preoccupation: measures the perception of intense separation anxiety and excessive current preoccupation with attachment figures (items 12, 14, 18, 26, 31, and 32). (3) Parental interference: measures childhood reminiscences of overprotection, the experience of fear, and preoccupation with abandonment (items 4, 20, 25, and 27). (4) Value of parental authority: measures positive perceptions of family values of authority and hierarchy (items 5, 19, and 29). (5) Parental permissiveness: measures memories of lack of parental limits and guidance during childhood (items 2, 15, and 22). (6) Self-sufficiency and resentment against parents: measures resentment towards parents and rejection of dependence and affective reciprocity (items 8, 9, 16, and 24). (7) Childhood trauma: measures childhood memories of lack of availability, violence, and threats by attachment figures (items 1, 10, 17, 23, and 28). The questionnaire has adequate reliability, with subscales Cronbach’s alpha ranging from 0.60 to 0.85.

#### 2.2.3. Problem-Oriented Screening Instrument for Teenagers (POSIT)

We used only the sections on substance use and mental health, comprising 37 items, out of the global scale that includes a total of 81 items [36]. In the substance use section, affirmative responses to the items are considered as risk indicators of substance misuse for adolescents. In the section regarding mental health, adolescents with five or more affirmative responses, or affirmative responses to any one of the items 6, 28, 55, 75, or 76, are considered at risk. The questionnaire has high reliability (alpha = 0.90). Besides, four separate items were included to obtain information regarding the frequency of current and past use of psychoactive substances.

#### 2.2.4. Center for Epidemiologic Studies of Depression Scale CESD-R

This revised version of the CES-D was created to map symptoms of depression to the DSM-IV major depressive episode construct. The scale has 35 items that assess the presence of depressive symptoms in the past two weeks (with five response options each: 0 days; 1–2 days; 3–5 days; 5–7 days; and 8–14 days). The scale has high reliability (alpha = 0.85) [37,38].

#### 2.2.5. Beck Anxiety Inventory BAI

This scale has 21 items that evaluate anxiety symptoms that do not overlap with depression during the past week. Responses are presented in a four-point Likert scale (1 = not at all; 2 = mildly; 3 = moderately; and 4 = severely), corresponding to values of zero through three. To determine the anxiety level, all responses were summed and the total score was interpreted as follows: between 0 and 5 points indicate a minimal level of anxiety; 6–15 points represent mild anxiety; a score of 16–30 points means moderate anxiety, and a score of 31–63 points indicates severe anxiety. The scale has high reliability (alpha = 0.83) [39,40].

#### 2.2.6. Beck’s Hopelessness Scale HS-UAA 18

The scale has 18 items that measure hopelessness, understood as a deep sense of having lost motivation, the possibility that good things may happen in the future, and the belief that adverse situations will change for the better. It identifies pessimism and negative attitudes toward the future, and the ability to overcome difficulties and to achieve success in life. The items have a dichotomous response (true or false) and a point is assigned to those indicating hopelessness. The rating ranges are: 0–3 = normal or asymptomatic; 4–8 = mild; 9–14 = moderate; and 15–20 = severe. A score equal to 9 or higher is an indicator of suicidal behavior. The scale has high reliability (KR = 0.92) [41,42].

#### 2.2.7. Trait Meta-Mood Scale TMMS-24

The scale includes 24 items that measure emotional intelligence, which is the ability to be aware of and regulate one’s emotions. Responses use a 5-point Likert scale (1 = totally disagree to 5 = totally agree). The scale has three subscales: (1) emotional attention measures the ability to feel and express emotions adequately; (2) emotional clarity measures the perception of the understanding of one’s emotional states, and (3) emotional repair measures the perceived ability to regulate one’s emotional states. A very low or very high score on emotional attention was considered as an indicator of difficulties in the ability to adequately feel and express emotions (very high = anxiety and depression and very low = limitations in social functioning). The scale has high reliability (alpha = 0.85–0.89) [43,44].

#### 2.2.8. Rosenberg Self-Esteem Scale

This scale includes 10 items that evaluate self-esteem, based on adolescents’ thoughts and feelings about themselves that contribute to their sense of personal worth and satisfaction in interpersonal interactions. The response format is a four-point Likert scale (1 = totally agree to 4 = totally disagree). The scale has adequate reliability (alpha = 0.79) [45,46].

#### 2.2.9. Sociodemographic Characteristics

Additionally, the questionnaire included a general information section to obtain sociodemographic characteristics, such as age, gender, school grade, and whether basic and non-basic economic needs were sufficiently met in the participant’s family.

### 2.3. Data Collection and Analysis Procedure

**Figure 1:**
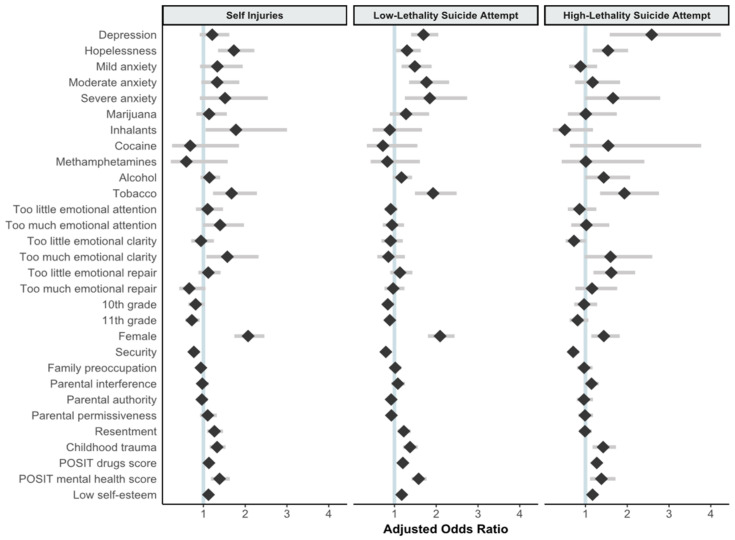
Adjusted odds ratios with 95% confidence intervals for the outcomes of self-injury, low-lethality suicide attempt, or high-lethality suicide attempt, relative to no suicidal behavior, by selected covariates.

Since the data were collected using a standardized questionnaire, data preparation followed the scoring instructions given for each scale or instrument as described above. Preliminary analysis was used to confirm the validity of scale scores, and psychometric characteristics of each instrument in the present sample. Bivariate analysis used frequencies, proportions and means to report sample characteristics. In accordance with the nominal measurement scale of the study’s outcome, we used multinomial logistic regression to assess the association of each variable with suicidal behavior. Unadjusted odds ratios (ORs) of suicidal behavior were estimated in bivariate regressions of the outcome with each covariate. Adjusted odds ratios (aORs) were estimated including all variables simultaneously in the model, presenting their 95% confidence intervals (CIs) and associated *p*-values. To facilitate the analysis of results, a Figure 1 illustrates all aORs and their CIs. In categorical variables, the aORs convey the difference in the odds comparing each group or category to a referent category (generally not being exposed to the characteristic), holding constant all other variables in the model. Since all continuous variables were nearly or normally distributed, the measures were centered around the mean and standardized to a standard deviation (sd) = 1. Hence, the interpretation of aORs indicates the increase or decrease in the odds of suicidal behavior associated with a one-sd difference in the covariate, holding all other variables constant. The analyses accommodate the structure of the sample recognizing potential lack of independence of observations across schools, and therefore providing corrected standard errors for the calculation of 95% CI and *p*-values. Finally, we adopted the conventional level of 0.05 as a threshold for statistical significance.

## 3. Results

Table 1 reports sample characteristics and bivariate analysis of suicide behavior on selected covariates. A larger number of females than males participated in the study (51.3% vs. 48.7%, respectively, *p* < 0.001). Additionally, a slightly higher proportion of students attending 1st and 3rd years of the high school participated in the survey, as compared to 5th-year students although the differences did not reach statistical significance (37.7%, 34.9%, and 27.4%, respectively, with *p* = 0.381). One in five students had suicidal behavior (20.5%; self-injury, 5.7%; low-lethality suicide attempt, 11.2%; and high-lethality suicide attempt, 3.6%).

Results from bivariate analyses were as follows. The majority of students did not have severe symptoms of depression (86.4%) or hopelessness (86.1%), but those who had were over-represented among those with suicidal behaviors (*p* < 0.001). A little more than half of the students had higher than minimal anxiety, with about one in four students having moderate or severe levels (20.1% and 5.3%, respectively); differences in anxiety level were associated with suicidal behavior (*p* < 0.001). The most widely used substance in the past year was alcohol (47.1%), followed by tobacco (19.1%) and marijuana (8.9%). Cocaine, inhalants, and methamphetamine use were reported also by about one in 20 students (5%, 4.9%, and 4.3%, respectively), and use of each of these substances was associated with suicidal behavior (*p* < 0.001).

Different domains of emotional intelligence were associated with suicidal behavior. Overall, most students’ emotional regulation through emotional attention was classified in either “Too little” (48.1%) or “Too much” (14.4%) categories; no clear statistical difference was observed associating emotional attention and suicidal behavior (*p* = 0.065). In contrast, a stronger statistical signal was observed for students’ emotional regulation through emotional clarity (*p* < 0.001) where those who scored in the “Too little” category had a higher proportion of suicidal behavior (6.5% for self-injuries, 13.6% for low-lethality suicide attempt, and 4.1% high-lethality suicide attempt). Similarly, emotional intelligence through emotional repair was strongly associated with suicidal behaviors, with a higher percentage of students in the categories of “Too little” (40.9%) and “Too low” (20.2%), and a similar pattern of association such that those with too little emotional repair resources had higher percentages of self-injuries (7.4%), low-lethality suicide attempt (15.2%), and high-lethality suicide attempt (5.2%).

The bottom third of Table 1 conveys data from continuous variables before centering them around the mean. The first set of variables belong to attachment domains. The score for all these variables ranged from 2 to 6 and their means varied between 3.3 (childhood trauma), 3.4 (parental permissiveness), 3.9 (resentment), 4.1 (parental interference), 4.2 (family preoccupation), 4.9 (security), to 5.1 (parental authority). The most notable differences were observed when comparing average scores between students with no suicidal behavior to their counterparts with low-lethality suicide attempts in the security domain (means = 4.9 and 4.3, respectively) and childhood trauma (means = 3.2 and 3.8, when comparing no suicidal behavior to low-lethality suicide attempt, respectively).

POSIT scores ranged from 0 to 17 for drug use problems and from 0 to 16 for mental health problems, with means of 0.7 and 5.2, respectively. However, higher scores were observed in both POSIT scales in association with suicidal behavior. Finally, low self-esteem scale scores ranged from 10 to 40 with a mean of 22.2. Higher scores of a low self-esteem were observed among youth with suicidal behavior (means = 24.3 for self-injuries, 24.9 for low-lethality suicide attempts, and 25.0 for high-lethality suicide attempts). Of note, all variables in this panel (i.e., measured on an interval scale) were statistically significant (*p* < 0.001).

The association between suicidal behavior and covariates is presented in Table 2. Unadjusted estimates show that students with depressive symptoms had almost five times the odds of self-injuries, eight times of a low-lethality suicide attempt, and almost ten times of a high-lethality suicide attempt than their counterparts without symptoms. However, when simultaneously controlling for all other variables in the model, the adjusted odds ratios (aORs) were lower albeit still highly statistically significant for a low-lethality suicide attempt (aOR= 1.69; 95% CI = 1.40–2.05; *p* < 0.001) and for a high-lethality suicide attempt (aOR = 2.59; 95% CI = 1.58–4.24; *p* < 0.001). Similarly, students with a high score on hopelessness had 3–4 times the odds of suicidal behavior than those with lower scores; controlling for all other variables in the model attenuated the strength of the association but did not eliminate statistical significance. Hence, the odds of self-injuries for those with hopelessness were 1.73 times the odds for those without it (95% CI = 1.35–2.22; *p* < 0.001), 1.30 for a low-lethality suicide attempt (95% CI = 1.04–1.63; *p* = 0.024), and 1.54 for a high-lethality suicide attempt (95% CI = 1.17–2.02; *p* = 0.002). Anxiety was found to be associated with suicidal behavior, with increased odds among those with higher levels of anxiety. For example, the unadjusted OR for self-injuries was 2.37 comparing those with mild vs. minimal anxiety, 3.75 when comparing moderate vs. minimal, and 6.40 for those with severe vs. minimal anxiety. The strength and precision of the estimated association weakened considerably in the multivariate regression in general terms, except for low-lethality suicide attempts. In fact, those with a mild level of anxiety had 1.49 times the adjusted odds of low-lethality suicide attempt compared to those with a minimal level (95% CI = 1.17–1.89; *p* < 0.001), 1.77 times the adjusted odds if the level of anxiety was moderate (95% CI = 1.35–2.31; *p* < 0.001), and 1.85 times for a severe level of anxiety (95% CI = 1.25–2.74; *p* = 0.003).

In unadjusted analyses, past-month drug use was moderately associated with suicidal behavior with ORs ranging from 1.34 for use of methamphetamines to 2.27 for tobacco use. Adjusting for covariates affected the strength of the association in most cases, except for tobacco use and the odds of high-lethality suicide attempt (aOR = 1.93; 95% CI = 1.35–2.76; *p* < 0.001).

Concerning emotional intelligence, those with inadequate responses to attention to emotions (i.e., too little or too much) had higher odds of suicidal behavior (OR = 1.34 and 1.62, respectively). Multivariate adjustment attenuated, even more, the association and weakened the statistical precision of estimates (*p* > 0.05). Focusing on emotional clarity was not found to be associated with suicidal behavior in multivariate analysis although the unadjusted estimates pointed to higher odds corresponding to too little or too much use of this strategy. Finally, there was a complex pattern of association between emotional repair and suicidal behavior, such that when the strategy score was too little the odds of a high-lethality suicide attempt were higher than when the score was deemed adequate (aOR = 1.62; 95% CI = 1.19–2.19; *p* = 0.002). On the other hand, despite the lack of statistical significance, when the score for use of emotional repair corresponded to “Too much” the odds of self-injuries were lower than those corresponding to “adequate” (aOR = 0.66; 95% CI = 0.42–1.05; *p* = 0.076).

The multivariate analyses indicated an inverse association between being in more advanced high-school grades and the odds of self-injury (aOR = 0.81; 95% CI= 0.64–1.03; *p* = 0.080 for 10th graders and aOR = 0.77; 95% CI = 0.57–0.91; *p* = 0.007 for 11 graders), and for a low-lethality suicide attempt (aOR = 0.84; 95% CI = 0.71–0.99; *p* = 0.043 for 3rd graders and aOR = 0.89; 95% CI = 0.77–1.02; *p* = 0.101 for 5th graders). Finally, a statistically robust association was observed between being female and having increased odds of each of the suicidal behaviors. For example, females had twice the odds of self-injuries compared to males (aOR = 2.07; 95% CI = 1.74–2.46; *p* < 0.001), low-lethality suicide behavior (aOR = 2.09; 95% CI = 1.80–2.44; *p* < 0.001), and about one and a half times the odds of a high-lethality suicide attempt (aOR = 1.44; 95% CI = 1.14–1.82; *p* = 0.003).

Some of the attachment dimensions were consistently associated with suicidal behavior as shown at the bottom panel of Table 2 referring to continuous variables. The security dimension was inversely associated with all types of suicidal behavior, both in the unadjusted and adjusted estimates. For example, a difference in one standard deviation in security was associated with lower odds of self-injury (OR = 0.62; aOR = 0.77; 95% CI = 0.66–0.90; *p* < 0.001), a low-lethality suicide attempt (OR = 0.61; aOR = 0.79; 95% CI = 0.71–0.89; *p* < 0.001), and a high-lethality suicide attempt (OR = 0.55; aOR = 0.71; 95% CI = 0.59–0.85; *p* < 0.001). A one-sd difference in resentment was associated with self-injuries and low-lethality suicide attempt (aOR = 1.26; 95% CI = 1.09–1.47; *p* = 0.003; and aOR = 1.22; 95% CI = 1.08–1.39; *p* = 0.003, respectively). Childhood trauma was directly associated with suicidal behaviors; a one-standard-deviation difference on the childhood trauma score was associated with higher odds of self-injuries (aOR = 1.33; 95% CI = 1.15–1.53; *p* < 0.001), a low-lethality suicide attempt (aOR = 1.37; 95% CI = 1.21–1.56; *p* < 0.001), and with a high-lethality suicide attempt (aOR = 1.42; 95% CI = 1.17–1.73; *p* < 0.001).

Higher scores on both POSIT scales were found to be consistently associated with suicidal behavior. A difference of one-sd on the drugs scale was associated with self-injuries (aOR = 1.13; 95% CI = 1.03–1.24; *p* = 0.010), a low-lethality suicide attempt (aOR = 1.20; 95% CI = 1.13–1.28; *p* < 0.001), and a high-lethality suicide attempt (aOR = 1.27; 95% CI = 1.18–1.37; *p* < 0.001). Similarly, the mental health score was also associated with suicidal behavior. A one-sd difference in the mental health score was associated with higher odds of self-injuries (aOR = 1.39; 95% CI = 1.18–1.63; *p* < 0.001), a low-lethality suicide attempt (aOR = 1.58; 95% CI = 1.41–1.77; *p* < 0.001), and a high-lethality suicide attempt (aOR = 1.38; 95% CI = 1.11–1.72; *p* = 0.004).

Finally, low self-esteem was also found to be consistently associated with suicidal behavior (aOR = 1.12; 95% CI = 1.02–1.23; *p* = 0.018), a low-lethality suicide attempt (aOR = 1.17; 95% CI = 1.06–1.30; *p* = 0.002), and a high-lethality suicide attempt (aOR = 1.17; 95% CI = 1.05–1.31; *p* = 0.006).

## 4. Discussion

The present study expanded prior research by introducing new constructs to a model that had been tested earlier in the context of a case-control study in one of the states with the highest suicide rates in Mexico [25], providing an opportune view of the current situation of young people residing under confinement during the COVID-19 pandemic. Almost 21% of all students reported suicidal behavior, 11% with a low-lethality suicide attempt, and close to 4% with a high-lethality suicide attempt. This is problematic, as one of the strongest indicators of completed suicide is having had prior attempts [4]. Of note is that close to 6% of young people in the present study reported self-inflicted injuries, which has been documented as an indicator of acute suffering [47], and as evidence of the progression of suicidal behavior [19]. Among people who survived a suicide attempt, follow-up studies report approximately 2% risk of suicide in the past 12 months, and approximately 4% risk of suicide in the past five years [48]. Unfortunately, the present data only provide a snapshot of the current situation and do not allow us to determine whether conditions have worsened or improved during the pandemic, for example, economic turmoil, social isolation, online education, even immune function, and inflammatory responses if infected by COVID-19. Nonetheless, a comparison to research elsewhere suggests that the current situation for Aguascalientes’ youth is no better and probably worse, as other studies have reported prevalence estimates between 9% and 20% [8,9,10,11].

All variables in bivariate analyses showed highly significant associations with suicidal behaviors. This is not surprising as covariate selection was steered through a conceptual lens and models and the evidence accrued from the scientific literature. The attenuation of unadjusted associations when multivariate adjustment was introduced was also expected given the inter-relation of many of the variables under study and the large number of covariates that were used simultaneously in the models. In this context, the main findings of the present study can be summarized as follows: (1) females had consistently higher odds of suicidal behavior compared to males; (2) depression, hopelessness, and anxiety were also independently and consistently associated with suicidal behavior; (3) severity of mental health problems were also independently and consistently associated with higher odds of suicidal behavior; (4) severity of drug use problems was also significantly associated with suicidal behaviors; independently from the severity of drug use problems, use of alcohol, and more clearly tobacco, was also consistently associated with suicidal behaviors; (5) childhood trauma and having to self-rely as issues affecting attachment were also found to be associated with suicidal behavior; (6) finally, while low self-esteem was associated with higher odds of suicidal behavior, security of attachment was associated with lower odds of suicidal behavior.

Before we discuss these findings, it is important to acknowledge that the present study has several important limitations that merit disclosure. First, population-based research, such as the present study, requires the use of survey research methods with large samples, screening instruments and probabilistic classification of outcomes and exposures, rather than the intensive case ascertainment available in the context of smaller-sample clinical studies. However, as noted in Section 2, all our measures have been shown to have adequate reliability and validity. Second, no pre-COVID-19 data had been collected for this specific population, which prevents the study from being able to assess whether and how the situation might have changed in the past year. Third, the data was self-reported by unsupervised students, which means that accuracy and precision are influenced by willingness and ability to report potentially stigmatized behaviors. Fourth, our statistical analysis could not fully operationalize the complexity of the conceptual model, leaving ample room for future analysis that may better accommodate the complex variable inter-relations. Additionally, even with a relatively large dataset, some suicidal behaviors have low frequencies creating small-cell issues for analyses with a large number of covariates.

Notwithstanding these and other limitations, the study helps identify specific challenges currently affecting adolescents. The literature provides several examples of studies where covariates are associated with suicidal behavior among young people, for example, depression and hopelessness [49,50,51,52], comorbidity between anxiety, depression, and suicidal behavior [53,54], and drug use [55,56,57,58,59]. An unexpected finding was the high proportion of students who reported using drugs, especially because estimates in the present study were much higher than the national averages for this group [60]. A point for further inquiry is how young people were able to gain access to and even use these drugs given the confinement regulations induced during the COVID-19 pandemic. Additionally, self-esteem has been consistently found to be associated with suicidal behavior [25,61]. Similar to this study, Johnson et al. [62], found that childhood trauma was associated with the risk of suicidal behavior in the adolescent population. This association is especially important since prior research has reported that childhood trauma is associated with an increase in depressive symptoms and anxiety traits [63], variables that had a strong association in our results. Van der Vegt et al. [64] documented an association between low levels of positive thinking about the future (hopelessness in our study) and suicidal behavior, and Nock et al. [65] found that anxiety and poor impulse control are significantly associated with suicidal ideation and suicide attempts. Regarding episodes of major depression, the study carried out by Arsenault-Lapierre et al. [66] suggested that this type of mental disorder is the most common in people who die by suicide. Finally, Arsenault-Lapierre et al. [66]; Dumais et al. [67]; Seguin et al. [68]; Artenie et al. [69] found significant associations between suicide risk and drug use.

The odds of self-harm and low-lethality suicide attempts were lower for adolescents in higher school grades who were therefore older. This is probably due to the maturation process and the acquisition of more skills [70]. Females reported a higher risk than males of being in a suicidal behavior group. This is consistent with the evidence and may point to gender elements that expose women to contend daily with psychosocial stressors that increase vulnerability [20,71].

Resentment and childhood trauma were associated with greater odds of belonging to any of the three risk groups, which is consistent with the literature regarding the association between these two variables, especially if sexual abuse was inflicted [72,73]. Childhood sexual abuse has been found to be strongly associated with suicidal behavior, especially with co-occurring anxiety [74,75,76].

Exploratory analysis of the current data showed that a higher number of problems was associated with the odds of suicidal behavior but we did not find a particular trend concerning the type or severity of the behavior (analyses not shown). Clearly, a more complex pattern of relationships between the variables is needed. The work of Turecki et al. [14] can illuminate how these factors contribute to suicide risk in adolescence and early adulthood as they posit that most models include the interplay of predisposing, precipitating, and developmental factors, showing the interaction between distal, developmental, and proximal factors that may lead to suicidal behaviors.

Placing the evidence in the context of these theoretical models, we surmised that suicidal behaviors among adolescents and young adults may be explained through a model of dispositional, mediating, and triggering-maintenance factors for which the unit of analysis is the individual in interaction within a sociohistorical context. Hence, suicidal behavior is conceptualized as the result of dysfunctional relationships between the individuals and their context. Dispositional factors account for the interactions that individuals have developed through their developmental lifespans, which influence the constellation of psychological resources to cope with negative emotions and potentially stressful situations, which are the mediating factors. This is why our analyses show higher odds of suicide among students with attachment issues and with emotional regulation deficits. The model suggests that failure to develop these psychological resources, coupled with exposure to stressful situations, increases the occurrence of high-risk behaviors (e.g., binge drinking or illegal drug use) and the emergence and/or aggravation of affective disorders. This creates feedback loops that constantly reinforce each other (as shown in the present study via higher scores on the POSIT for drug and mental health-related problems), generating a desire to die and, ultimately, leading to suicide. The model considers the fact that depression is a risk factor found in most studies, indicating that the comorbidity of depressive disorders with anxiety and problematic drug involvement strongly suggests failed attempts to deal with stress and psychological pain in the context of inadequate coping resources.

## 5. Conclusions

Having no pre-pandemic comparable data, it might be difficult to evaluate how life might have changed for adolescents in Aguascalientes, Mexico. However, the evidence reported here highlights several areas where adolescents face tremendous developmental and wellbeing challenges, with a high risk of suicidal behaviors. These challenges might have been exacerbated during the COVID-19 pandemic. As vaccines are added to public health measures to contain and reduce the risk of infection and attenuate its impact on individuals and communities, we surmise comprehensive efforts are urgently needed as well to address the psychosocial needs of young people.

## Figures and Tables

**Table 1:** Sample characteristics and bivariate analysis of suicide behavior on selected covariates. Data from the study “Variables Psicosociales Implicadas en el Desarrollo de Adolescentes y Jóvenes, VIDA-J [Psychosocial Variables Implicated in Adolescents’ and Youth’s Development]” in Aguascalientes, Mexico (*n* = 8033).

Variable	Total	Sample %	No Suicidal Behavior (*n* = 6390, 79.5%)		Self-Injuries (*n* = 454, 5.7%)		Low-Lethality Suicide Attempt (*n* = 897, 11.2%)		High-Lethality Suicide Attempt (*n* = 292, 3.6%)	
			*n*	wt % **	*n*	wt % **	*n*	wt % **	*n*	wt % **
Sex			Design-based F(2.85, 176.69) = 108.0410 *p* < 0.001							
Male	3910	48.7	3401	87.0	146	3.7	250	6.4	113	2.9
Female	4123	51.3	2989	72.5	308	7.5	647	15.7	179	4.3
High-School Grade			Design-based F(5.32, 329.93) = 1.0650 *p* = 0.3810							
9th	3029	37.7	2380	78.6	191	6.3	349	11.5	109	3.6
10th	2805	34.9	2238	79.8	154	5.5	302	10.8	111	4.0
11th	2199	27.4	1772	80.6	109	5.0	246	11.2	72	3.3
Depression			Design-based F(2.70, 167.55) = 305.7240 *p* < 0.001							
No	6938	86.4	5910	85.2	327	4.7	539	7.8	162	2.3
Yes	1095	13.6	480	43.8	127	11.6	358	32.7	130	11.9
Hopelessness			Design-based F(2.71, 167.88) = 87.7198 *p* < 0.001							
No	6919	86.1	5748	83.1	324	4.7	649	9.4	198	2.9
Yes	1114	13.9	642	57.6	130	11.7	248	22.3	94	8.4
Anxiety (BAI)			Design-based F(6.80, 421.36) = 90.7285 *p* < 0.001							
Minimal	3682	45.8	3335	90.6	118	3.2	155	4.2	74	2.0
Mild	2314	28.8	1813	78.4	152	6.6	280	12.1	69	3.0
Moderate	1615	20.1	1039	64.3	138	8.5	340	21.1	98	6.1
Severe	422	5.3	203	48.1	46	10.9	122	28.9	51	12.1
Past Month Use										
Marijuana			Design-based F(2.40, 149.05) = 30.0118 *p* < 0.001							
No	7321	91.1	5926	81.0	395	5.4	761	10.4	239	3.3
Yes	712	8.9	464	65.2	59	8.3	136	19.1	53	7.4
Inhalants			Design-based F(2.23, 138.04) = 8.4606 *p* < 0.001							
No	7639	95.1	6110	80.0	420	5.5	843	11.0	266	3.5
Yes	394	4.9	280	71.1	34	8.6	54	13.7	26	6.6
Cocaine			Design-based F(2.61, 161.92) = 10.7100 *p* < 0.001							
No	7634	95.0	6106	80.0	425	5.6	844	11.1	259	3.4
Yes	399	5.0	284	71.2	29	7.3	53	13.3	33	8.3
Methamphetamines			Design-based F(2.63, 163.20) = 7.3596 *p* < 0.001							
No	7685	95.7	6135	79.8	430	5.6	853	11.1	267	3.5
Yes	348	4.3	255	73.3	24	6.9	44	12.6	25	7.2
Alcohol			Design-based F(2.48, 153.64) = 64.1876 *p* < 0.001							
No	4249	52.9	3632	85.5	190	4.5	333	7.8	94	2.2
Yes	3784	47.1	2758	72.9	264	7.0	564	14.9	198	5.2
Tobacco			Design-based F(2.54, 157.18) = 90.9564 *p* < 0.001							
No	6502	80.9	5412	83.2	322	5.0	590	9.1	178	2.7
Yes	1531	19.1	978	63.9	132	8.6	307	20.1	114	7.5
Emotional Intelligence										
Emotional attention			Design-based F(4.84, 300.14) = 2.1194 *p* = 0.065							
Adequate	3013	37.5	2444	81.1	139	4.6	330	11.0	100	3.3
Too little	3864	48.1	3056	79.1	233	6.0	436	11.3	139	3.6
Too much	1156	14.4	890	77.0	82	7.1	131	11.3	53	4.6
Emotional clarity			Design-based F(4.33, 268.15) = 16.3275 *p* < 0.001							
Adequate	2380	29.6	1989	83.6	103	4.3	220	9.2	68	2.9
Too little	4471	55.7	3388	75.8	292	6.5	609	13.6	182	4.1
Too much	1182	14.7	1013	85.7	59	5.0	68	5.8	42	3.6
Emotional repair			Design-based F(4.91, 304.19) = 36.8942 *p* < 0.001							
Adequate	3124	38.9	2608	83.5	154	4.9	288	9.2	74	2.4
Too little	3286	40.9	2372	72.2	244	7.4	498	15.2	172	5.2
Too much	1623	20.2	1410	86.9	56	3.5	111	6.8	46	2.8
**Variable**	**Total**	**Mean**	**Mean**	**95% CI**	**Mean**	**95% CI**	**Mean**	**95% CI**	**Mean**	**95% CI**
Attachment *										
Security	8033	4.9	4.9	4.9, 5.0	4.4	4.3, 4.5	4.4	4.3, 4.4	4.3	4.0, 4.3
Family preoccupation	8033	4.2	4.2	4.2, 4.3	4.2	4.1, 4.3	4.3	4.3, 4.4	4.2	4.0, 4.3
Parental interference	8033	4.1	4.1	4.1, 4.1	4.2	4.1, 4.3	4.3	4.2, 4.4	4.2	4.1, 4.3
Parental authority	8033	5.1	5.2	5.1, 5.2	4.9	4.8, 5.0	5.0	4.9, 5.0	4.8	4.7, 4.9
Parental permissiveness	8033	3.4	3.4	3.4, 3.4	3.5	3.4, 3.6	3.5	3.4, 3.5	3.5	3.3, 3.6
Resentment	8033	3.9	3.8	3.8, 3.8	4.2	4.1, 4.3	4.3	4.3, 4.4	4.1	5.0, 4.3
Childhood trauma	8033	3.3	3.2	3.1, 3.2	3.7	3.6, 3.8	3.8	3.7, 3.8	3.7	3.6, 3.8
POSIT *										
Drugs	8033	0.7	0.4	0.5, 0.5	1.2	1.0, 1.4	1.6	1.4, 1.7	2.0	1.7, 2.4
Mental health	8033	5.2	4.4	4.3, 4.5	7.8	7.4, 8.3	8.9	8.7, 9.2	8.5	7.9, 9.2
Low self-esteem *	8033	22.2	21.6	21.4, 21.7	24.3	23.9, 24.8	24.9	24.3, 25.7	25.0	24.4, 25.7

* Denotes a continuous variable, standardized with mean = 0 and standard deviation = 1; all statistically significant with *p* ≤ 0.001; ** wt % refers to the 95% confidence interval. The estimates accommodate the complex survey design; statistical tests account for design effect.

**Table 2:** Results of multinomial logistic regression of suicide behavior on selected covariates. Data from the study “Variables Psicosociales Implicadas en el Desarrollo de Adolescentes y Jóvenes, VIDA-J [Psychosocial Variables Implicated in Adolescents’ and Youth’s Development]” in Aguascalientes, Mexico (*n* = 8033).

Variable	Self-Injuries						Low-Lethality Suicide Attempt				High-Lethality Suicide Attempt				
	OR	aOR	*p*	95% CI		OR	aOR	*p*	95% CI		OR	aOR	*p*	95% CI	
				ll	uL				ll	uL				ll	uL
Depression															
No	1.00		(referent)			1.00	1.00	(referent)			1.00	1.00	(referent)		
Yes	4.78	1.21	0.193	0.91	1.62	8.17	1.69	<0.001	1.40	2.05	9.88	2.59	<0.001	1.58	4.24
Hopelessness															
No	1.00	1.00	(referent)			1.00	1.00	(referent)			1.00	1.00	(referent)		
Yes	3.59	1.73	<0.001	1.35	2.22	3.42	1.30	0.024	1.04	1.63	4.25	1.54	0.002	1.17	2.02
Anxiety (BAI)															
Minimal	1.00	1.00	(referent)			1.00	1.00	(referent)			1.00	1.00	(referent)		
Mild	2.37	1.33	0.129	0.92	1.94	3.32	1.49	0.001	1.17	1.89	1.72	0.88	0.503	0.61	1.28
Moderate	3.75	1.33	0.096	0.95	1.86	7.04	1.77	<0.001	1.35	2.31	4.25	1.17	0.478	0.75	1.83
Severe	6.40	1.52	0.111	0.91	2.54	12.93	1.85	0.003	1.25	2.74	11.32	1.66	0.056	0.99	2.79
Past Month Use															
Marijuana															
No	1.00	1.00	(referent)			1.00	1.00	(referent)			1.00	1.00	(referent)		
Yes	1.91	1.14	0.427	0.83	1.56	2.28	1.28	0.179	0.89	1.83	2.83	1.01	0.981	0.58	1.75
Inhalants															
No	1.00	1.00	(referent)			1.00	1.00	(referent)			1.00	1.00	(referent)		
Yes	1.77	1.78	0.032	1.05	3.00	1.4	0.89	0.708	0.48	1.66	2.13	0.51	0.114	0.22	1.18
Cocaine															
No	1.00	1.00	(referent)			1.00	1.00	(referent)			1.00	1.00	(referent)		
Yes	1.47	0.68	0.449	0.25	1.85	1.35	0.72	0.400	0.34	1.55	2.74	1.55	0.334	0.63	3.77
Methamphetamines															
No	1.00	1.00	(referent)			1.00	1.00				1.00	1.00	(referent)		
Yes	1.34	0.59	0.291	0.22	1.58	1.24	0.83	0.570	0.43	1.61	2.25	1.01	0.977	0.43	2.41
Alcohol															
No	1.00	1.00	(referent)			1.00	1.00	(referent)			1.00	1.00	(referent)		
Yes	1.83	1.14	0.192	0.93	1.40	2.23	1.17	0.121	0.96	1.42	2.77	1.44	0.053	0.99	2.07
Tobacco															
No	1.00	1.00	(referent)			1.00	1.00	(referent)			1.00	1.00	(referent)		
Yes	2.27	1.67	0.001	1.23	2.28	2.88	1.92	< 0.001	1.49	2.49	3.54	1.93	< 0.001	1.35	2.76
Emotional intelligence															
Emotional attention															
Adequate	1.00	1.00	(referent)			1.00	1.00	(referent)			1.00	1.00	(referent)		
Too little	1.34	1.10	0.509	0.82	1.47	1.06	0.91	0.265	0.77	1.07	1.11	0.86	0.424	0.58	1.26
Too much	1.62	1.40	0.053	0.99	1.97	1.09	0.94	0.656	0.72	1.23	1.46	1.02	0.925	0.66	1.57
Emotional clarity															
Adequate	1.00	1.00	(referent)			1.00	1.00	(referent)			1.00	1.00	(referent)		
Too little	1.66	0.94	0.666	0.71	1.25	1.63	0.91	0.487	0.69	1.20	1.57	0.73	0.059	0.52	1.01
Too much	1.12	1.57	0.023	1.07	2.32	0.61	0.86	0.410	0.59	1.25	1.21	1.60	0.058	0.98	2.60
Emotional repair															
Adequate	1.00	1.00	(referent)			1.00	1.00	(referent)			1.00	1.00	(referent)		
Too little	1.74	1.12	0.355	0.88	1.41	1.90	1.13	0.288	0.90	1.43	2.56	1.62	0.002	1.19	2.19
Too much	0.67	0.66	0.076	0.42	1.05	0.71	0.97	0.799	0.76	1.24	1.15	1.16	0.491	0.76	1.76
High-School Grade															
9th	1.00	1.00	(referent)			1.00	1.00	(referent)			1.00	1.00	(referent)		
10th	0.86	0.81	0.080	0.64	1.03	0.92	0.84	0.043	0.71	0.99	1.08	0.97	0.813	0.73	1.28
11th	0.77	0.72	0.007	0.57	0.91	0.95	0.89	0.101	0.77	1.02	0.89	0.81	0.134	0.62	1.07
Sex															
Male	1.00	1.00	(referent)			1.00	1.00	(referent)			1.00	1.00	(referent)		
Female	2.40	2.07	<0.001	1.74	2.46	2.94	2.09	<0.001	1.80	2.44	1.80	1.44	0.003	1.14	1.82
Attachment *															
Security	0.62	0.77	0.001	0.66	0.90	0.61	0.79	<0.001	0.71	0.89	0.55	0.71	<0.001	0.59	0.85
Family preoccupation	1.00	0.94	0.276	0.84	1.05	1.16	1.02	0.770	0.89	1.16	0.94	0.97	0.726	0.80	1.17
Parental interference	1.15	0.97	0.710	0.84	1.13	1.32	1.08	0.287	0.93	1.25	1.14	1.15	0.066	0.99	1.32
Parental authority	0.80	0.96	0.579	0.84	1.10	0.83	0.92	0.184	0.82	1.04	0.73	0.96	0.712	0.79	1.18
Parental permissiveness	1.22	1.10	0.277	0.92	1.32	1.1	0.93	0.165	0.83	1.03	1.09	0.99	0.891	0.83	1.18
Resentment	1.79	1.26	0.003	1.09	1.47	2.04	1.22	0.003	1.08	1.39	1.59	0.99	0.868	0.85	1.15
Childhood trauma	1.93	1.33	<0.001	1.15	1.53	2.12	1.37	<0.001	1.21	1.56	2.02	1.42	0.001	1.17	1.73
POSIT *															
Drugs	1.44	1.13	0.010	1.03	1.24	1.56	1.20	<0.001	1.13	1.28	1.69	1.27	<0.001	1.18	1.37
Mental health	2.34	1.39	<0.001	1.18	1.63	3.06	1.58	<0.001	1.41	1.77	2.77	1.38	0.004	1.11	1.72
Low self-esteem *	1.56	1.12	0.018	1.02	1.23	1.7	1.17	0.002	1.06	1.30	1.72	1.17	0.006	1.05	1.31

* Denotes a continuous variable, standardized with mean = 0 and standard deviation = 1; the estimates accommodate the complex survey design; statistical tests account for design effect; OR denotes “Odds Ratio”; aOR denotes “Adjusted OR”; 95% CI denotes “95% Confidence Interval”; the nominal base category is “No suicide behavior”.

## Data Availability

Data are not publicly available due to restrictions imposed at informed consent with participating institutions.

## References

[1] Pierce M., Hope H., Ford T., Hatch S., Hotopf M., John A., Kontopantelis E., Webb R., Wessely S., McManus S. (2020). Mental health before and during the COVID-19 pandemic: A longitudinal probability sample survey of the UK population. Lancet Psychiatry.

[2] Iob E., Steptoe A., Fancourt D. (2020). Abuse, self-harm and suicidal ideation in the UK during the COVID-19 pandemic. Br. J. Psychiatry.

[3] John A., Okolie C., Eyles E., Webb R.T., Schmidt L., McGuiness L.A., Olorisade B.K., Arensman E., Hawton K., Kapur N. (2020). The impact of the COVID-19 pandemic on self-harm and suicidal behaviour: A living systematic review. F1000Research.

[4] World Health Organization (2014). Preventing Suicide: A Global Imperative. https://www.who.int/mental_health/suicide-prevention/world_report_2014/en/.

[5] Franklin J.C., Ribeiro J.D., Fox K.R., Bentley K.H., Kleiman E.M., Huang X., Musacchio K.M., Jaroszewski A.C., Chang B.P., Nock M.K. (2017). Risk factors for suicidal thoughts and behaviors: A meta-analysis of 50 years of research. Psychol. Bull..

[6] Pollock N.J. (2019). Place, the Built Environment, and Means Restriction in Suicide Prevention. Int. J. Environ. Res. Public Health.

[7] John A., Pirkis J., Gunnell D., Appleby L., Morrissey J. (2020). Trends in suicide during the covid-19 pandemic. BMJ.

[8] Ivey-Stephenson A.Z., Demissie Z., Crosby A.E., Stone D.M., Gaylor E., Wilkins N., Lowry R., Brown M. (2020). Suicidal Ideation and Behaviors Among High School Students—Youth Risk Behavior Survey, United States, 2019. MMWR Suppl..

[9] Khan M.A., Rahman M., Islam R., Karim M., Hasan M., Jesmin S.S. (2020). Suicidal behavior among school-going adolescents in Bangladesh: Findings of the global school-based student health survey. Soc. Psychiatry Psychiatr. Epidemiol..

[10] Soto-Sanz V., Piqueras J.A., García-Olcina M., Rivera-Riquelme M., Rodríguez-Marín J., Alonso J. (2020). Relación Entre Conducta Suicida Y Síntomas Interiorizados En Niños Y Adolescentes. Psicol. Conduct..

[11] Uddin R., Burton N.W., Maple M., Khan S.R., Khan A. (2019). Suicidal ideation, suicide planning, and suicide attempts among adolescents in 59 low-income and middle-income countries: A population-based study. Lancet Child Adolesc. Health.

[12] World Health Organization (2019). Suicide in the World: Global Health Estimates. https://apps.who.int/iris/handle/10665/326948.

[13] World Health Organization SDG Target 3.4, Indicator 3.4.2 Suicide Mortality Rate. https://unstats.un.org/sdgs/metadata?Text=&Goal=3&Target=3.4.

[14] Turecki G., Brent D.A., Gunnell D., O’Connor R.C., Oquendo M.A., Pirkis J., Stanley B.H. (2019). Suicide and suicide risk. Nat. Rev. Dis. Prim..

[15] Cha C.B., Franz P.J., Guzmán E.M., Glenn C.R., Kleiman E.M., Nock M.K. (2017). Annual Research Review: Suicide among youth—epidemiology, (potential) etiology, and treatment. J. Child Psychol. Psychiatry.

[16] Chang H.B., Munroe S., Gray K., Porta G., Douaihy A., Marsland A., Brent D., Melhem N.M. (2019). The role of substance use, smoking, and inflammation in risk for suicidal behavior. J. Affect. Disord..

[17] Conejero I., Nobile B., Olié E., Courtet P. (2021). How Does COVID-19 Affect the Neurobiology of Suicide?. Curr. Psychiatry Rep..

[18] Glenn C.R., Lanzillo E.C., Esposito E.C., Santee A.C., Nock M.K., Auerbach R.P. (2017). Examining the Course of Suicidal and Nonsuicidal Self-Injurious Thoughts and Behaviors in Outpatient and Inpatient Adolescents. J. Abnorm. Child Psychol..

[19] Nock M.K., Green J.G., Hwang I., McLaughlin K.A., Sampson N.A., Zaslavsky A.M., Kessler R.C. (2013). Prevalence, Correlates, and Treatment of Lifetime Suicidal Behavior Among Adolescents. JAMA Psychiatry.

[20] Miranda-Mendizabal A., Castellví P., Parés-Badell O., Alayo I., Almenara J., Alonso I., Blasco M.J., Cebrià A., Gabilondo A., Gili M. (2019). Gender differences in suicidal behavior in adolescents and young adults: Systematic review and meta-analysis of longitudinal studies. Int. J. Public Health.

[21] Instituto Nacional de Estadística y Geografía Defunciones por Suicidio por Entidad Federativa de Residencia Habitual de la Persona Fallecida Según Sexo, 2010 a 2019. https://www.inegi.org.mx/app/tabulados/interactivos/?pxq=Mortalidad_Mortalidad_07_8627c147-473c-4c63-9967-56664e612f40.

[22] Instituto Nacional de Estadística y Geografía Estadísticas a Propósito del día Mundial Para la Prevención del Suicidio: Datos Nacionales. Comunicado de Prensa INEGI. https://www.inegi.org.mx/contenidos/saladeprensa/aproposito/2020/suicidios2020_Nal.pdf.

[23] Instituto Nacional de Estadística y Geografía Estadísticas a Propósito del día Mundial de la Prevenció del Suicidio (10 DE SEPTIEMBRE): Datos Nacionales. Comunicado de Prensa 455/19. https://www.inegi.org.mx/contenidos/saladeprensa/aproposito/2019/suicidios2019_Nal.pdf.

[24] Reger M.A., Stanley I.H., Joiner T.E. (2020). Suicide Mortality and Coronavirus Disease 2019—A Perfect Storm?. JAMA Psychiatry.

[25] Hermosillo-De-La-Torre A.E., González-Forteza C., Rivera-Heredia M.E., Méndez-Sánchez C., González-Betanzos F., Palacios-Salas P., Jiménez A., Wagner F.A. (2020). Understanding suicidal behavior and its prevention among youth and young adults in Mexico. Prev. Med..

[26] The World Medical Association WMA Declaration of Helsinki-Ethical Principles for Medical Research Involving Human Subjects. https://www.wma.net/policies-post/wma-declaration-of-helsinki-ethical-principles-for-medical-research-involving-human-subjects/.

[27] González-Forteza C., Juárez-López C.E., Jiménez A., Montejo-León L., Rodríguez-Santisbón U.R., Wagner F.A. (2017). Suicide behavior and associated psychosocial factors among adolescents in Campeche, Mexico. Prev. Med..

[28] González-Forteza C., Ramos L.L., Caballero M.Á.G., Wagner F.A.E. (2003). Correlatos psicosociales de depresión, ideación e intento suicida en adolescentes mexicanos. Psicothema.

[29] Gonzalez-Forteza C., Alvarez-Ruiz M., Saldaña-Hernández A., Carreño-García S., Chávez-Hernández A.-M., Pérez-Hernández R. (2005). PREVALENCE OF DELIBERATE SELF-HARM IN TEENAGE STUDENTS IN THE STATE OF GUANAJUATO, MEXICO: 2003. Soc. Behav. Pers. Int. J..

[30] Díez-Gómez A., Pérez-Albéniz A., Sebastián-Enesco C., Fonseca-Pedrero E. (2020). Suicidal Behavior in Adolescents: A Latent Class Analysis. Int. J. Environ. Res. Public Health.

[31] Arenas-Monreal L., Hidalgo-Solórzano E., Chong-Escudero X., la Cruz J.A.D., González-Cruz N.L., Pérez-Matus S., Valdez-Santiago R. (2021). Suicidal behaviour in adolescents: Educational interventions in Mexico. Health Soc. Care Community.

[32] Balluerka N., Lacasa F., Gorostiaga A., Muela A., Pierrehumbert B. (2011). Versión reducida del cuestionario CaMir (CaMir-R) para la evaluación del apego. Psicothema.

[33] Pierrehumbert B., Karmaniola A., Sieye A., Meister C., Miljkovitch R., Halfon O. (1996). Les modèles de relations: Développement d’un autoquestionnaire d’attachement pour adultes. Psychiatr. l’Enfant.

[34] Main M., Cassidy J., Shaver P.R. (1999). Epilogue. Attachment theory: Eighteen points with suggestions for future studies. Handbook of Attachment: Theory, Research, and Clinical Applications.

[35] Lacasa F., Muela A. (2014). Guía para la aplicación e interpretación del cuestionario de apego CaMir-R. Rev. Psicopatol. Salud Ment. Niño Adolesc..

[36] Mariño M.D.C., González-Forteza C., Andrade P., Medina-Mora M.E. (1998). Validación de un cuestionario para detectar adolescentes con problemas por el uso de drogas. Salud Ment..

[37] Eaton W.W., Smith C., Ybarra M., Muntaner C., Tien A. (2004). Center for Epidemiologic Studies Depression Scale: Review and Revision (CESD and CESD-R). The Use of Psychological Testing for Treatment Planning and Outcomes Assessment: Instruments for Adults.

[38] González-Forteza C., Jiménez-Tapia J.A., Ramos-Lira L., Wagner F.A. (2008). Aplicación de la Escala de Depresión del Center of Epidemiological Studies en adolescentes de la Ciudad de México. Salud Pública México.

[39] Robles R., Varela R., Jurado S., Páez F. (2001). The Mexican version of Beck Anxiety Inventory: Psychometric properties. Rev. Mex. Psicol..

[40] Beck A.T., Epstein N., Brown G., Steer R.A. (1988). An inventory for measuring clinical anxiety: Psychometric properties. J. Consult. Clin. Psychol..

[41] La Torre A.E.H.-D., Méndez-Sánchez C., Gonzalez-Betanzos F. (2020). Evidencias de validez factorial de la Escala de desesperanza de Beck en español con muestras clínicas y no clínicas. Acta Colomb. Psicol..

[42] Beck A.T., Weissman A., Lester D., Trexler L. (1974). The measurement of pessimism: The Hopelessness Scale. J. Consult. Clin. Psychol..

[43] Fernandez-Berrocal P., Extremera N., Ramos N. (2004). Validity and Reliability of the Spanish Modified Version of the Trait Meta-Mood Scale. Psychol. Rep..

[44] Salovey P., Mayer J.D., Goldman S.L., Turvey C., Palfai T.P., Pennebaker J.W. (1995). Emotional attention, clarity and repair: Exploring emotional intelligence using the Trait Meta-Mood Scale. Emotion, Disclosure and Health.

[45] Rosenberg M. (1965). Society and the Adolescent Self-Image.

[46] Jiménez Tapia A., Mondragón Barrios L., González-Forteza C. (2007). Self-esteem, depressive symptomatology, and suicidal ideation in adolescents: Results of three studies. Salud Ment..

[47] Sánchez-Teruel D., Robles-Bello M.A., Camacho-Conde J.A. (2020). Self-inflicted injuries in adolescents and young adults: A longitudinal approach. Psicothema.

[48] Carroll R., Metcalfe C., Gunnell D. (2014). Hospital Presenting Self-Harm and Risk of Fatal and Non-Fatal Repetition: Systematic Review and Meta-Analysis. PLoS ONE.

[49] Benjet C., Borges G., Medina-Mora M.E., Zambrano J., Aguilar-Gaxiola S. (2009). Youth mental health in a populous city of the developing world: Results from the Mexican Adolescent Mental Health Survey. J. Child Psychol. Psychiatry.

[50] Gili M., Castellví P., Vives M., de la Torre-Luque A., Almenara J., Blasco M.J., Cebrià A.I., Gabilondo A., Pérez-Ara M.A., Miranda-Mendizábal A. (2019). Mental disorders as risk factors for suicidal behavior in young people: A meta-analysis and systematic review of longitudinal studies. J. Affect. Disord..

[51] Rodríguez S.P., Salvador J.H.M., García-Alandete J. (2017). The role of hopelessness and meaning in life in a clinical sample with non-suicidal self-injury and suicide attempts. Psicothema.

[52] Wolfe K.L., Nakonezny P.A., Owen V.J., Rial K.V., Bs A.P.M., Kennard B.D., Emslie G.J. (2019). Hopelessness as a Predictor of Suicide Ideation in Depressed Male and Female Adolescent Youth. Suicide Life-Threat. Behav..

[53] De La Vega D., Giner L., Courtet P. (2018). Suicidality in Subjects With Anxiety or Obsessive-Compulsive and Related Disorders: Recent Advances. Curr. Psychiatry Rep..

[54] Herres J., Shearer A., Kodish T., Kim B., Wang S.B., Diamond G.S. (2019). Differences in Suicide Risk Severity Among Suicidal Youth With Anxiety Disorders. Crisis.

[55] Borges G., Benjet C., Orozco R., Medina-Mora M.-E., Menendez D. (2017). Alcohol, cannabis and other drugs and subsequent suicide ideation and attempt among young Mexicans. J. Psychiatr. Res..

[56] Kim Y.J., Burlaka V. (2017). Gender Differences in Suicidal Behaviors: Mediation Role of Psychological Distress between Alcohol Abuse/Dependence and Suicidal Behaviors. Arch. Suicide Res..

[57] Poorolajal J., Darvishi N. (2016). Smoking and Suicide: A Meta-Analysis. PLoS ONE.

[58] Lange S., Koyanagi A., Rehm J., Roerecke M., Carvalho A.F. (2019). Association of Tobacco Use and Exposure to Secondhand Smoke With Suicide Attempts Among Adolescents: Findings From 33 Countries. Nicotine Tob. Res..

[59] Orri M., Galera C., Turecki G., Forte A., Renaud J., Boivin M., Tremblay R.E., Côté S.M., Geoffroy M.-C. (2018). Association of Childhood Irritability and Depressive/Anxious Mood Profiles with Adolescent Suicidal Ideation and Attempts. JAMA Psychiatry.

[60] Villatoro Velázquez J.A., Medina-Mora Icaza M.E., Hernández Ávila M., Instituto Nacional de Psiquiatría de la Fuente Muñiz, Instituto Nacional de Salud Pública, Comisión Nacional Contra las Adicciones, Secretaría de Salud (2017). Encuesta Nacional de Consumo de Drogas, Alcohol y Tabaco 2016–2017: Reporte de Alcohol.

[61] Soto-Sanz V., Piqueras J.A., Rodríguez-Marín J., Pérez-Vázquez M., Rodríguez-Jiménez T., Castellví P., Miranda-Mendizábal A., Parés-Badell O., Almenara J., Blanco M.J. (2019). Self-esteem and suicidal behaviour in youth: A meta-analysis of longitudinal studies. Psicothema.

[62] Johnson J.G., Cohen P., Gould M.S., Kasen S., Brown J., Brook J.S. (2002). Childhood Adversities, Interpersonal Difficulties, and Risk for Suicide Attempts During Late Adolescence and Early Adulthood. Arch. Gen. Psychiatry.

[63] Roy A., Gorodetsky E., Yuan Q., Goldman D., Enoch M.-A. (2010). Interaction of FKBP5, a Stress-Related Gene, with Childhood Trauma Increases the Risk for Attempting Suicide. Neuropsychopharmacology.

[64] Van Der Vegt E.J.M., Van Der Ende J., Ferdinand R.F., Verhulst F.C., Tiemeier H. (2008). Early Childhood Adversities and Trajectories of Psychiatric Problems in Adoptees: Evidence for Long Lasting Effects. J. Abnorm. Child Psychol..

[65] Nock M.K., Hwang I., Sampson N., Kessler R.C., Angermeyer M., Beautrais A., Borges G., Bromet E., Bruffaerts R., De Girolamo G. (2009). Cross-National Analysis of the Associations among Mental Disorders and Suicidal Behavior: Findings from the WHO World Mental Health Surveys. PLoS Med..

[66] Arsenault-Lapierre G., Kim C., Turecki G. (2004). Psychiatric diagnoses in 3275 suicides: A meta-analysis. BMC Psychiatry.

[67] Dumais A., Lesage A., Alda M., Rouleau G., Dumont M., Chawky N., Roy M., Mann J., Benkelfat C., Turecki G. (2005). Risk Factors for Suicide Completion in Major Depression: A Case-Control Study of Impulsive and Aggressive Behaviors in Men. Am. J. Psychiatry.

[68] Séguin M., Lesage A., Turecki G., Bouchard M., Chawky N., Tremblay N., Daigle F., Guy A. (2007). Life trajectories and burden of adversity: Mapping the developmental profiles of suicide mortality. Psychol. Med..

[69] Artenie A.A., Bruneau J., Roy É., Zang G., Lespérance F., Renaud J., Tremblay J., Jutras-Aswad D. (2015). Licit and illicit substance use among people who inject drugs and the association with subsequent suicidal attempt. Addiction.

[70] Li C.-Q., Zhang J.-S., Ma S., Lv R.-R., Duan J.-L., Luo D.-M., Yan X.-J., Ma N., Song Y. (2020). Gender differences in self-harm and drinking behaviors among high school students in Beijing, China. BMC Public Health.

[71] Vijayakumar L. (2015). Suicide in women. Indian J. Psychiatry.

[72] Goldsmith S.K., Pellmar T.C., Kleinman A.M., Bunney W.E. (2002). Reducing Suicide: A National Imperative.

[73] Restrepo D.M., Chesin M.S., Jeglic E.L. (2016). The Relationship between Social Maladjustment, Childhood Abuse and Suicidal Behavior in College Students. Int. J. Psychol. Psychol. Ther..

[74] Sheftall A.H., Asti L., Horowitz L.M., Felts A., Fontanella C.A., Campo J.V., Bridge J.A. (2016). Suicide in Elementary School-Aged Children and Early Adolescents. Pediatrics.

[75] Cassels M., Baetens I., Wilkinson P., Hoppenbrouwers K., Wiersema J.R., Van Leeuwen K., Kiekens G. (2019). Attachment and Non-Suicidal Self-Injury among Young Adolescents: The Indirect Role of Behavioral Problems. Arch. Suicide Res..

[76] Davaji R.B.O., Valizadeh S., Nikamal M. (2010). The relationship between attachment styles and suicide ideation: The study of Turkmen students, Iran. Procedia Soc. Behav. Sci..

